# Supplementary material for: The Chemical Profile, and Antidermatophytic, Anti-Inflammatory, Antioxidant and Antitumor Activities of Withania chevalieri A.E. Gonç. Ethanolic Extract
Source: Plants (Basel). 2023 Jun 30;12(13):2502. doi: 10.3390/plants12132502 (PMC10347035; doi:10.3390/plants12132502)
Supplement: Supplementary file 1 [file plants-12-02502-s001.zip › plants-2394449-supplementary.pdf]

Supplemental Materials

Viability of human skin keratynocytes (HaCat) cell line exposed to *Withania chevalieri* extract (WcCEE)

Background

Since the ethanolic extract of *Withania chevalieri* (WcCEE) is used in skin diseases, including skin infections by dermatophytes, we studied the viability of a human skin keratynocytes cell line (Ha Cat cells) when exposed to WcCEE.

Materials and Methods

The cytotoxic potential of the WcCEE extract on Human Skin Keratinocytes (HaCat) cell line was also quantified. Briefly, early-passage HaCat cell line cultures (Germany Cancer Research Center - DKFZ, Heidelberg, Germany) were grown as adherent cultures using cell culture bottles with DMEM, containing 10% FBS with phenol red, and incubated under standard conditions (37 °C, 5% CO_2, 95% air) in a humidified incubator. Cell suspensions were prepared to obtain a final concentration of $1X10^5$ cells/well in a 96-well microdilution plate. After an incubation of 24 h, the culture medium was removed and 200 µL of DMEM (without phenol red) containing different concentrations of the extract were added into each well. All the plates were incubated for 24 h. After incubation, 20 µL of MTS (3-(4,5-Dimethylthiazol-2-yl)-5-(3-carboxymethoxyphenyl)-2-(4-sulfophenyl)-2H-tetrazolium) at 2 mg/mL was added and the plates were further incubated during 4 h. Each experiment included a non-treated group as the control. Cell viability was assessed based on spectrophotometric (Spectramax ID3) analysis at 490 nm. All data from this study are representative of at least three independent and triplicate experiments. The cell viability was expressed as the percentage of viable cells when compared with controls.

RESULTS

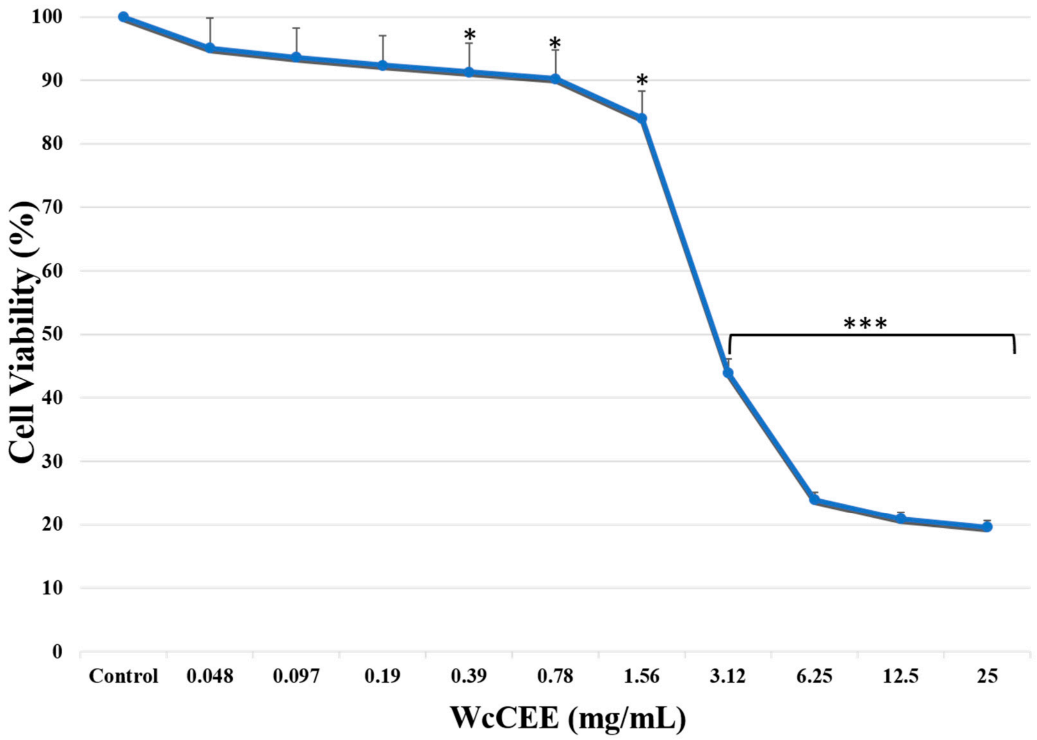

Figure S1: HaCat Viability Dose-response Curve. HaCat cells were treated with different concentrations of WcCEE ranging from 0.048 to 25 mg/mL. DMEM was used as a negative control. Cell viability was measured by MTS assay and the results were given as means ± SD (n = 3) of cell viability after exposition to WcCEE for 24h. * indicate statistically significantly different from control. *P* values of (*$P \leq 0.05$) (**$P \leq 0.01$) are considered significant.
